# Assessment of knowledge, attitudes, and practices on vaccine usage among small ruminant farmers in the Northern Region of Bangladesh

**DOI:** 10.14202/vetworld.2024.1435-1448

**Published:** 2024-07-06

**Authors:** Md. Sodrul Islam, Apurbo Kumar Mondal, Md. Rabiul Auwul, Tahrima Islam, Obaidul Islam, Afroja Yasmin, Md. Abdullah Al Mahmud, A.K.M. Ziaul Haque, Mahmuda Begum, Jahid Hasan Tipu, Ysharzya Mojumder, Manna Roy, Md. Ashraful Islam

**Affiliations:** 1 Department of Physiology and Pharmacology, Faculty of Veterinary Medicine and Animal Science, Bangabandhu Sheikh Mujibur Rahman Agricultural University, Gazipur, Bangladesh; 2 Department of Statistics, Faculty of Agricultural Economics and Rural Development, Bangabandhu Sheikh Mujibur Rahman Agricultural University, Gazipur, Bangladesh; 3 Laboratory of Veterinary Epidemiology, College of Veterinary Medicine, Chungbuk National University, Cheongju, South Korea; 4 Department of Pathobiology, Faculty of Veterinary Medicine and Animal Science, Bangabandhu Sheikh Mujibur Rahman Agricultural University, Gazipur, Bangladesh; 5 Department of Anatomy and Histology, Faculty of Veterinary Medicine and Animal Science, Bangabandhu Sheikh Mujibur Rahman Agricultural University, Gazipur, Bangladesh; 6 Kazi Farms Poultry Laboratory, Gazipur, Bangladesh; 7 Department of Livestock Production and Management, Sylhet Agricultural University, Sylhet, Bangladesh; 8 Department of Global Public Health and Primary Care, University of Bergen, Norway; 9 Faculty of Veterinary, Animal and Biomedical Sciences, Sylhet Agricultural University, Sylhet, Bangladesh; 10 Department of Pharmacology and Toxicology, Sylhet Agricultural University, Sylhet, Bangladesh; 11 Department of Livestock Services, Ministry of Fisheries and Livestock, Dhaka, Bangladesh; 12 Laboratory of Veterinary Laboratory Medicine, College of Veterinary Medicine, Chungbuk National University, Cheongju, South Korea

**Keywords:** Bangladesh, knowledge, attitudes, and practices, small ruminant farmers, vaccine use

## Abstract

**Background and Aim:**

Small ruminants require vaccines to prevent and manage diseases. Unfortunately, no studies have been conducted in Bangladesh to assess the knowledge, attitudes, and practices (KAP) of small ruminant farmers (SRF) regarding vaccine use against infectious diseases, affecting the success of vaccination campaigns. The present study aims to assess SRF’s KAP regarding vaccines, revealing gaps and barriers to efficient vaccination.

**Materials and Methods:**

Two hundred and twenty-eight SRF in northern Bangladesh were surveyed in a cross-sectional study. Data were collected from random participants through face-to-face interviews using a structured questionnaire. KAP levels were categorized as “good” or “poor” and “positive” or “negative” using a scoring method with a 60% cutoff. The analysis comprised the utilization of descriptive statistics as well as logistic regression models.

**Results:**

Results showed that most participants were female (60.5%), aged 31–40 (34.2%), with secondary education (28.1%), and vaccination training (22.8%). While 75% knew about vaccines, only 37.3% understood their role in preventing infectious diseases, and 63.6% in reducing antibiotic use 68.4% of farmers were aware of negative drawbacks, and 61.8% reported vaccinating their herds. About 42.1% of the farmers had good knowledge, 52.6% had a positive attitude, and 22.8% followed good practices. Female farmers with graduate degrees and 6–10 years of goat farming experience, but not those with vaccination training, demonstrated stronger knowledge. Female farmers with a graduate degree and 6–10 years of goat farming experience displayed positive attitudes. Female goat farmers from Thakurgaon had a higher likelihood of following good vaccination practices than those with vaccination training.

**Conclusion:**

The study unearths disparities in KAP scores among farmers. To effectively address KAP gaps concerning vaccine usage and prevent potential infectious diseases, it is essential to design focused educational and training programs. About 52.6% of SRF hold a positive view toward vaccines.

## Introduction

In Bangladesh, livestock contributes 16.52% to agricultural GDP and 1.85% to the overall economy [1]. Around 10% of annual animal deaths are due to diseases that worsen when biosecurity and vaccination are insufficient [2]. About 36% of daily animal protein intake comes from livestock, impacting 6 million lives [3, 4]. Sheep and goats, called the “cow of the poor,” significantly contribute to Bangladesh’s rural economy, raised mainly in backyard systems by impoverished women and children [3, 5]. Traditional management practices in rural areas heighten the vulnerability of sheep and goats to several diseases and injuries, causing significant economic losses and reduced productivity [5]. Approximately 10% of animals die annually from these illnesses, which are exacerbated by inadequate biosecurity and vaccination [2].

Vaccines for small ruminants not only safeguard consumers and boost their productivity but also provide an economically viable solution, reducing mortality rates and enhancing overall health [6]. Administering vaccines promptly and managing health effectively are suggested ways to prevent (the development of) illnesses [7, 8]. The financial and psychological factors that impact household decisions regarding vaccines are yet to be explained [8, 9]. Zoonotic pathogens pose greater health risks to both humans and animals in underdeveloped settings [10]. Small ruminant vaccines not only protect consumers and enhance small ruminant productivity but also contribute to a favorable cost-benefit ratio by reducing mortality rates and improving overall health [6]. Amidst rising antibiotic resistance, vaccination assumes significant importance for managing human and small ruminant health [11]. Innovative livestock vaccinations are essential to meet the demand for chemical-free food during concerns about drug residues in meat, eggs, and milk continue to rise [6].

Effective immunization strategies rely on producers’ knowledge, attitudes, and practices (KAP) for optimal vaccine benefits and longevity [12]. In Ethiopia’s Amhara region, farmers’ limited understanding of diseases and vaccines contributes to low vaccination rates [13]. Farmers’ ignorance regarding vaccine storage, handling, and delivery is a concern [14]. Factors including cost, accessibility, and cultural beliefs shape people’s vaccination attitudes [9, 15]. Research shows disparities in livestock farmers’ adherence to recommended vaccination schedules and methods [14, 16]. Enhancing vaccination coverage and minimizing wastage necessitate extra training on vaccine management.

In Bangladesh, a significant majority of farmers (85.7%) rely on unqualified individuals for small ruminant treatment, with only 14.3% consulting veterinarians [5]. Although Peste des Petits Ruminants (PPR) is the most commonly reported illness among small ruminant farmers (SRF), only 16.3% vaccinate their goats and sheep against it [5]. The previous study by Sivachandiran *et al*. [17] found that 22% of farmers vaccinated their goats; 49% were vaccinated against PPR, 25% were vaccinated against PPR and Anthrax [17 18], and 57.33% 26% were vaccinated against key diseases such as goat pox and PPR [19]. Developing successful vaccination strategies necessitates an understanding of farmers’ KAP toward vaccines [12, 20, 21]. In Bangladesh, there is a paucity of research on the efficacy of vaccines for different livestock species.

This study is the first to assess SRF’s KAP regarding vaccines, revealing gaps and obstacles to effective vaccination.

## Materials and Methods

### Ethical approval and Informed consent

The study protocol, assigned reference number FVMAS/AREC/2023/7, was approved by the Animal Research Ethics Committee (AREC) of Bangabandhu Sheikh Mujibur Rahman Agricultural University after a thorough evaluation. All participants gave verbal consent, guaranteeing their voluntary involvement, the protection of their rights, as well as the privacy of their information. The safety and well-being of research participants were prioritized throughout the ethical approval and consent procedures.

### Study period and location

**Figure-1:**
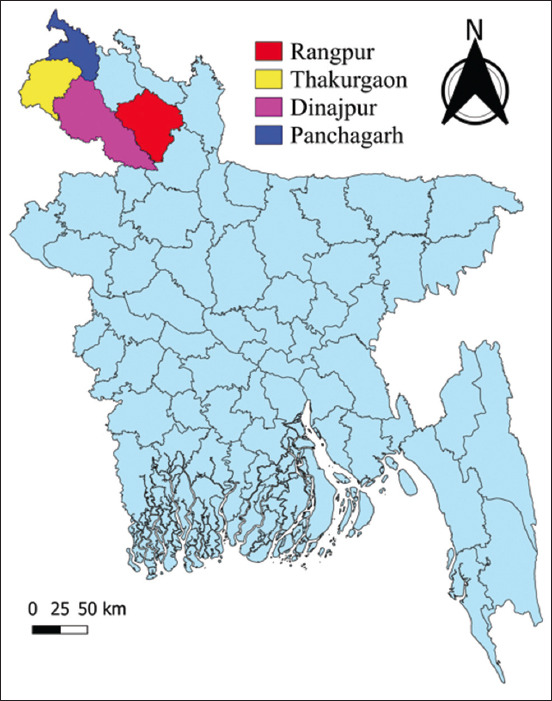
The map visually displays the study region in Bangladesh, with surveys represented by various colors in selected districts. QGIS version 3.14 (http://qgis.org) was used to generate this map.

The study was conducted for 6 months, from July to December 2023, in four districts of the northern region of Bangladesh, namely, Rangpur, Panchagarh, Thakurgaon, and Dinajpur (Figure-1). A total of 16 upazilas (a district’s lowest administrative border) were surveyed within the northern region, with four from each district. The selection of these districts was based on data supplied by the relevant District Livestock Office, specifically focusing on the highest density of small ruminant farms.

### Study design

Based on the Raosoft sample size calculation method [22], this study employed a sample size. 98 goat farmers, 60 sheep farmers, and 70 mixed farmers contributed a total of 228 farmers to the information gathering process. The farmer ensures that the daily needs of farm animals were met through feeding, watering, and care. The participant should be an 18-year-old or older individual with a connection to the farm. Farmers were selected from a list provided by the Upazila Livestock Offices based on their willingness to participate in the study. Randomly selected individuals from the group ensured sample representation. With a 5% margin of error, 85% confidence level, and a 50% response rate distribution, the results were analyzed. A 5% non-response rate was taken into account [22]. A 50% sample proportion was selected due to the scarcity of comparable research in the chosen location. 196 was the minimum required sample size for this study. Two hundred and twenty-eight participants were recruited to bolster the study’s robustness. A predesigned paper questionnaire was used to gather the required data. Two hundred and twenty-eight participating farmers comprised the groups with 60 each from Rangpur (Rangpur Sadar, Pirgacha, Mithapukur, and Pirganj), 60 from Panchagarh (Panchagarh Sadar, Debiganj, Boda, and Atwari), 56 from Thakurgaon (Thakurgaon Sadar, Baliadangi, Ranisankail, and Haripur), and 52 from Dinajpur (Dinajpur Sadar, Phulbari, Nawabganj, and Ghoraghat). Thirteen farmers were interviewed in each upazila during the survey.

### Design of questionnaires and collection of data

The questionnaire used in this study consisted of four sections (A to D). The section (A) included demographic information such as age, gender, education, district, category of farm, duration of farming experience, and training related to vaccines. Both the knowledge (B) and attitude (C) sections consisted of 13 distinct close-ended questions (K1–K13 for knowledge and A1–A12 for attitudes). The practice section (D) contained 12 questions (P1–P12 for practices), some of which were closed-ended and others were open-ended. The questionnaire was pre-tested with 20 SRF and revised accordingly. Data were collected by veterinarians and veterinary students from the participants through face-to-face interviews, which took approximately 45 min.

### Management of data, scoring, and statistical analysis

The collected data were entered into Microsoft Excel (2016) file (Microsoft Office, Washington, USA), then cleaned, and processed for analysis. The KAP levels of the participants were evaluated using a scoring system. Responses were assigned a score of one for correct answers and 0 for incorrect answers. The correct answers to each question were aggregated to obtain a participant’s total score in each KAP domain. Thus, according to the structure of the questionnaire and scoring system, the maximum possible scores were 13, 13, and 12 for KAP, respectively. The percentage of a participant’s score was calculated by dividing his/her total score in each KAP domain with the maximum possible score of that KAP domain, then multiplied by 100. Following the analysis, the data were partitioned into two categories depending on the proportion of correct answers to KAP level questions. A cutoff point of ≥60% was used to determine good knowledge and good practice levels. Participants scoring >60% were having good attitudes [23]. Those who scored below this threshold were categorized as possessing poor knowledge, negative attitudes, and poor practices. In addition, Spearman’s rank correlation coefficient was employed to examine the correlation between the KAP scores.

### Statistical analysis

Statistical analysis was performed using IBM SPSS Statistics for Windows (Version 26.0) (IBM Corp., Armonk, NY, USA). We employed descriptive statistics to analyze the categorical variables, including frequency and percentage. With a significance level of p < 0.05, we used both univariate and multivariate analyses to investigate the relationships between dependent variables (KAP) and independent variables (sociodemographic). Univariate logistic regression was used to calculate the odds ratio (OR) along with the 95% confidence interval (CI) for the various sociodemographic variables. Following the screening process, only univariate variables with p ≤ 0.05 were incorporated in the final multivariate analysis. To perform multivariate logistic regression analysis, we used the backward elimination method. The final multivariate logistic regression model was then used to determine the adjusted OR (AOR) and 95% CIs. All statistical significance was determined at p = 0.05, and the findings were reported as AORs and 95% CIs. The Hosmer-Lemeshow evaluation was used to measure the appropriateness of the final KAP approaches. QGIS version 3.14 (http://qgis.org) was utilized to generate a map of the study area, and the radar chart was created using the fms package in R 4.3.2 (https://cran.r-project.org/web/packages/fmsb/index.html).

## Results

### Sociodemographic characteristics of farmers

We conducted a cross-sectional study involving 228 SRF in four districts of the Northern regions, namely, Rangpur, Panchagarh, Thakurgaon, and Dinajpur. Participant demographics and interview responses are detailed in Table-1. Among the 228 SRF, the majority were female (60.5%, n = 138/228), spanning a wide age range, particularly in the 31–40 years group (34.2%, n = 78/228) and the 41–50 years group (31.6%, n = 72/228). Participants exhibited diverse educational backgrounds, with 18.4% (n = 42/228) lacking formal education and 28.1% (n = 64/228) possessing secondary education degrees. The study included nearly equal representation from the four districts (Rangpur-26.3%, n = 60/228; Panchagarh-26.3%, n = 60/228; Thakurgaon-24.6%, n = 56/228; and Dinajpur-22.8%, n = 52/228). Goat farms accounted for 43.0% (n = 98/228), sheep farms for 26.3% (n = 60/228), and mixed farms for 30.7% (n = 70/228). Farming experience levels varied, with the majority (36.8%, n = 84/228) having over 15 years of experience. However, only 22.8% (n = 52/228) had received training on vaccine utilization, indicating a gap in vaccine expertise and procedures (Table-1).

**Table-1:** Sociodemographic characteristics of small ruminant farmers (n = 228) in the study area.

Variables	Category	Frequency (number)	Percentage
Gender	Male	90	39.5
	Female	138	60.5
Age	18–30 years	34	14.9
	31–40 years	78	34.2
	41–50 years	72	31.6
	≥50 years	44	19.3
Education	No formal education	42	18.4
	Primary	44	19.3
	Secondary	64	28.1
	Higher secondary	48	21.1
	Graduation and above	30	13.1
District	Rangpur	60	26.3
	Panchagarh	60	26.3
	Thakurgaon	56	24.6
	Dinajpur	52	22.8
Type of farm	Goat	98	43.0
	Mixed	70	30.7
	Sheep	60	26.3
Farming experience	1–5 years	38	16.7
	6–10 years	30	13.2
	11–15 years	76	33.3
	>15 years	84	36.8
Training on livestock diseases and vaccination	Received	52	22.8
	Not received	176	77.2

### Knowledge of farmers regarding vaccine use

The findings indicate that a majority (75.0%, n = 171/228) of SRFs are familiar with vaccines. In addition, more than half of the SRF (59.6%, n = 136/228) know illnesses affecting small ruminants, and 64.9% (n = 148/228) are aware of the history of previous illnesses on farms. Concerning beliefs about vaccines, 39.5% (n = 90/228) of SRF believed in their effectiveness, while a smaller proportion (41.7%, n = 95/228) know priority vaccinations. Moreover, a significant portion (74.6%, n = 170/228) express doubt about vaccines’ ability to prevent uncommon illnesses and question (38.6%, n = 88/228) the necessity of non-vaccine disease prevention methods. Noteworthy concerns about vaccines include 32.9% (n = 75/228) agreeing that some vaccines are better than others, and 68.4% (n = 156/228) expressing worries about potential negative effects. Understanding vaccination benefits is limited, with only 29.8% (n = 68/228) acknowledging these benefits and 37.3% (n = 85/228) understanding the importance of vaccinations in preventing zoonotic disease transmission. Moreover, a considerable number of individuals (66.7%, n = 152/228) express scepticism regarding the effectiveness of routine vaccinations in reducing antibiotic resistance, whereas 33.3% (n = 76/228) have doubts about their effectiveness. In addition, 41.2% (n = 94/228) recognize that some large ruminant illnesses can only be managed through vaccination (Table-2). Significant differences (p < 0.05) in each knowledge parameter of vaccine usage were observed among the SRF (Table-2).

**Table-2:** Assessment of participants’ knowledge level regarding the usage of vaccines for small ruminant diseases.

Variables	Categories	Frequency (n = 228)	Proportion (%)	p-value
K1. Have you heard of small ruminants’ vaccines?				
	Yes	171	75.0	0.000
	No	57	25.0%
K2. Understanding of small ruminants’ illnesses.				
	Yes	136	59.6	0.004
	No	92	40.4
K3. History of previous illnesses on the farm				
	Yes	148	64.9	0.000
	No	80	35.1
K4. Should vaccines effectively prevent small ruminants’ diseases?				
	Yes	90	39.5	0.001
	No	138	60.5
K5. Knowledge about priority small ruminants’ vaccination				
	Yes	95	41.7	0.012
	No	133	58.3
K6. Vaccines prevent uncommon diseases that do not affect your small ruminants				
	Yes	170	74.6	0.000
	No	58	25.4
K7. Should large ruminants’ diseases be restricted and prevented without vaccination?				
	Yes	88	38.6	0.001
	No	140	61.4
K8. Some small ruminants’ vaccines are more effective than others				
	Yes	75	32.9	0.000
	No	153	67.1
K9. Vaccination may have negative effects on small ruminants’ health.				
	Yes	156	68.4	0.000
	No	72	31.6
K10. Understanding the benefits of small ruminants’ vaccination				
	Yes	68	29.8	0.000
	No	160	70.2
K11. Small ruminants’ vaccination effectively prevents infectious disease transmission				
	Yes	85	37.3	0.000
	No	143	62.7
K12. Can routine vaccination mitigate antibiotic resistance issues in small ruminants?				
	Yes	76	33.3	0.000
	No	152	66.7
K13. Do you know several small ruminants’ diseases have no remedies other than vaccination?				
	Yes	94	41.2	0.008
	No	134	58.8	

### Attitudes of farmers regarding vaccine use

A significant percentage of SRF expressed disagreement regarding the easy availability of vaccines for small ruminant illnesses (50.9%, n = 116/228) and the belief that a single vaccine offers lifelong immunity (61.8%, n = 141/228) or equal protection against all diseases (59.2%, n = 135/228). However, 30.3% (n = 69/228) of SRF agreed that all herds should be prevented, even if some animals were not vaccinated, indicating a moderate understanding of herd immunity concepts. The majority of livestock farmers perceive vaccines as more costly than other disease prevention methods (82.4%, n = 165/228). Nevertheless, there is strong consensus on the necessity of vaccines for enhancing productivity and welfare (42.1%, n = 96/228), the significance of highly effective vaccines (60.7%, n = 148/228), as well as their importance in reducing antibiotic use (63.6%, n = 145/228). There is also notable agreement that healthy ruminants are less likely to get sick if vaccinated (65.8%, n = 150/228) and that vaccines are generally safe for both humans and animals (69.3%, n = 158/228). However, opinions vary on government funding for vaccines, with a majority opposing it (64.5%, n = 147/228), as well as on the role of vaccines in ensuring food safety, where disagreements are noticeable (60.1%, n = 137/228). SRF also differs on the impact of vaccinations on sustainable small ruminant farming (62.3%, n = 142/228) (Table-3). Similar to knowledge, each attitude level varied significantly among the SRF regarding vaccine use, except for the A1 variable (p < 0.05) (Table-3).

**Table-3:** Assessment of participants’ attitude level regarding the usage of vaccines for small ruminant diseases.

Variables	Categories	Frequency (n = 228)	Proportion (%)	p-value
A1. Vaccines for small ruminants’ diseases are easily available				
	Agree	116	50.9	0.791
	Disagree	112	49.1
A2. Do you think a single vaccine gives a small ruminants lifelong immunity?				
	Agree	87	38.2
	Disagree	141	61.8	
A3. Should one vaccine provide equal protection against all small ruminant’s diseases?				
	Agree	93	40.8	0.005
	Disagree	135	59.2	
A4. Small ruminants’ vaccines are more expensive than other disease preventive methods				
	Agree	165	82.4	0.000
	Disagree	63	27.6	
A5. Should all herds be protected if some small ruminants are vaccinated, and some are not?				
	Agree	69	30.3	0.000
	Disagree	159	69.7	
A6. The government should allocate funding for small ruminants’ vaccines				
	Agree	81	35.5	0.000
	Disagree	147	64.5	
A7. Vaccination can reduce the need for antibiotics in small ruminants				
	Agree	145	63.6	0.003
	Disagree	83	36.4	
A8. Use of vaccine is necessary to improve the productivity and welfare of small ruminants				
	Agree	96	42.1	0.017
	Disagree	132	57.9	
A9. A highly effective vaccine is important				
	Agree	148	60.7	0.000
	Disagree	80	35.08	
A10.Healthy small ruminants are less likely to get sick if given vaccine				
	Agree	150	65.8	0.000
	Disagree	78	34.2	
A11. Vaccines are generally used in human and animals because it is safe				
	Agree	158	69.3	0.000
	Disagree	70	30.7	
A12. Large ruminants’ vaccination makes our food safer.				
	Agree	91	39.9	0.002
	Disagree	137	60.1	
A13. Vaccination increases the sustainability of small ruminants farming				
	Agree	86	37.7	0.000
	Disagree	142	62.3	

### Practice of farmers regarding vaccine use

Most farmers (61.8%, n = 141/228) reported vaccinating their small ruminant herds, primarily in response to disease outbreaks (35.1%, n = 80/228). Notably, a significant proportion (56.6%, n = 129/228) of farmers do not keep vaccination records or follow a routine vaccination schedule (59.6%, n = 136/228). Furthermore, a notable portion (39.0%, n = 89/228) of participants mentioned the unavailability of vaccines for certain illnesses. In addition, most SRF (57.0%, n = 130/228) rely on veterinarian prescriptions when buying vaccines and take the time to review the vaccine prospectus (69.7%, n = 159/228). Concerning storage practices, the majority (62.7%, n = 143/228) use specific refrigerators for vaccines. However, it is alarming that a high proportion of SRF (63.2%, n = 144/228) do not check vaccine expiration dates or dispose of used or expired vials correctly (75.9%, n = 173/228). The data also revealed that a substantial portion of SRF (66.2%, n = 151/228) reported improper vaccination practices and a history of vaccine failure (35.1%, n = 80/228) (Table-4). Similar to the knowledge, each practice level varied significantly among the SRF regarding vaccine use (p < 0.05) (Table-4).

**Table-4:** Assessment of participants’ practice level regarding the usage of vaccines for small ruminant diseases.

Variables	Categories	Frequency (n = 228)	Proportion (%)	p-value
P1. Do you usually vaccinate your small ruminant herds?				
	Yes	141	61.8	0.000
	No	87	38.2
P2. When you vaccinate your small ruminants?				
	After outbreak of diseases	80	35.1	0.008
	Fellow farmer advice	54	23.7	
	Veterinarian recommendation	44	19.3	
	As per vaccination date and time	50	21.9	
P3. Do you keep a record of small ruminants’ vaccination you used earlier on the farm?				
	Yes	99	43.4	0.047
	No	129	56.6
P4. Do you follow any routine vaccination schedule in your small ruminant’s farm?				
	Yes	92	40.4	0.004
	No	136	59.6
P5. Are there any small ruminants’ illnesses for which vaccines are now unavailable?				
	Yes	89	39.0	0.001
	No	139	61.0
P6. Do you purchase small ruminants’ vaccines based on a veterinarian exact prescription?				
	Yes	130	57.0	0.034
	No	98	43.0
P7. Do you read the prospectus before administering small ruminants’ vaccines?				
	Yes	159	69.7	0.000
	No	69	30.3
P8. Where do you store your vaccines?				
	Specific refrigerator only for small ruminants’ vaccine	143	62.7	0.000
	Multipurpose refrigerator	75	32.9	
	Non-refrigerated cabinet	7	3.1	
	Others	3	1.3
P9. Do you check vaccine expiration dates before giving them to small ruminants?				
	Yes	84	36.8	0.000
	No	144	63.2
P10. Do you properly dispose of used or expired small ruminants’ vaccine vials and bottles?				
	Yes	55	24.1	0.000
	No	173	75.9
P11. Have you vaccinated small ruminants properly?				
	Yes	77	33.8	0.000
	No	151	66.2
P12. Is there a history of vaccine failure in your small ruminant’s farm?				
	Yes	80	35.1	0.000
	No	148	64.9	

### Factors affecting farmers’ KAP regarding vaccine use

#### Knowledge of farmers

**Figure-2:**
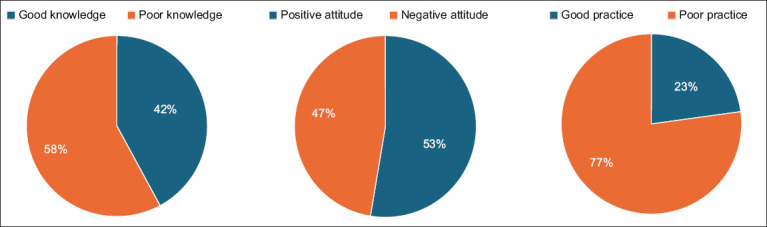
Knowledge, attitude, and practice of small ruminant farmers towards vaccine use.

This investigation revealed an overall good knowledge score of 42.1% (96/228) (Figure-2a). Univariate analysis showed significant associations (p < 0 .05) between participants knowledge levels and their gender, age, educational status, farm type, farming experience, and vaccine training. Female farmers were found to have 4.71 times greater odds of possessing good knowledge about vaccine use than males. Similarly, farmers aged 31–40 years were more likely to have good knowledge (OR: 4.50; 95% CI: 1.75–11.55) compared with those in the 18–30 age group. Furthermore, farmers with higher secondary education demonstrated significantly higher levels of good knowledge (OR: 8.40; 95% CI: 2.97–23.70) compared to those without any formal education. Goat farmers also exhibited significantly higher levels of good knowledge (OR: 5.62; 95% CI: 2.57–12.40) compared to sheep farmers. Surprisingly, farmers with 6–10 years (OR: 6.29; 95% CI: 2.32–17.68) as well as 11–15 years (OR: 6.60; 95% CI: 2.32–18.72) of experience performed better in the knowledge arena compared with those with a tenure of 1–5 years. However, farmers who received vaccination training from any organization were 76% less likely (OR: 0.24; 95% CI: 0.11–0.52) to have good knowledge than their non-trained counterparts. Nevertheless, districts did not show significant variability in this study (Table-5). Multivariate analysis revealed that knowledge levels of SRF significantly varied according to gender (p = 0.000), level of education (p = 0.001), farm category (p = 0.000), farming experience (p = 0.022), and vaccine training (p = 0.016) (Table-5).

**Table-5:** Multivariable and univariable analyses demonstrating the relationship between demographic variables and the level of knowledge, n = 228.

Variables	Knowledge level		Univariable analyses		Multivariable analyses	
		
	Good (%)	Poor (%)	OR (95% CI)	p-value	AOR (95% CI)	p-value

Gender						
Female	77	61	4.71 (2.56-8.66)	0.000	5.04 (2.35-10.79)	0.000
Male	19	71	Ref.	Ref.
Age						
31–40 years	42	36	4.50 (1.75–11.55)	0.006	2.45 (0.32–18.26)	0.077
41–50 years	33	39	3.26 (1.26–8.45)		0.23 (0.02–2.20)	
≥50 years	14	30	1.80 (0.63–5.12)		0.31 (0.02–3.72)	
18–30 years	7	27	Ref.	Ref.
Education						
Primary	16	28	3.42 (1.18–9.89)	0.001	2.08 (0.39–10.97)	0.001
Secondary	34	30	6.80 (2.51–18.37)		5.72 (1.44–22.68)	
Higher secondary	28	20	8.40 (2.97–23.70)		11.98 (3.06–46.86)	
Graduation and above	12	18	4.00 (1.29–12.40)		10.68 (1.30–87.73)	
No formal education	6	36	Ref.	Ref.	
District						
Rangpur	21	39	0.53 (0.25–1.150)	0.455		
Panchagarh	26	34	0.76 (0.36–1.61)			
Thakurgaon	23	33	0.69 (0.25–1.150)			
Dinajpur	26	26	Ref.
Farm type						
Goat	52	46	5.62 (2.57–12.40)	0.000	8.27 (3.12–21.87)	0.000
Mixed	34	36	4.72 (2.06–10.77)		6.03 (2.18–16.65)	
Sheep	10	50	Ref.		Ref.	
Experience in farming						
6–10 years	12	18	6.29 (2.23–17.68)	0.003	4.44 (0.67–29.36)	0.022
11–15 years	18	38	6.60 (2.32–18.72)		3.74 (2.94–36.67)	
>15 years	38	43	4.40 (1.33–14.47)		2.74 (2.73–26.42)	
1–5 years	5	33	Ref.		Ref.	
Training on livestock diseases and vaccination						
Received	10	42	0.24 (0.11–0.52)	0.000	0.30 (0.11–0.80)	0.016
Not received	86	90	Ref.		Ref.	

CI: confidence interval; OR: odd ratio; AOR: Adjusted odd ratio; Ref: reference category

#### Attitude of farmers

This investigation revealed an overall favorable attitude score of 52.6% (120/228) (Figure-2b). Univariate analysis demonstrated significant associations (p < 0.05) between higher attitude scores and factors such as age, gender, educational status, farm type, and farming experience. Female farmers exhibited 2.72 times greater odds of possessing a positive attitude than males. Similarly, farmers aged 31–40 years were more likely to have a positive attitude (OR: 8.00; 95% CI: 3.23–19.80) compared to those in the 18–30 age group. In addition, farmers who had completed primary schooling showed significantly higher levels of positive attitude (OR: 8.21; 95% CI: 3.05–22.13) compared to those without any formal education. Goat farmers also demonstrated significantly higher levels of positive attitude (OR: 6.18; 95% CI: 2.98–12.81) compared to sheep farmers. Surprisingly, farmers with more than 15 years of experience performed better (OR: 17.06; 95% CI: 6.24–46.67) in the attitude arena than those with a tenure of 1–5 years (Table-6). Multivariate analysis revealed that the attitude level of SRF significantly varied according to gender (p = 0.014), level of education (p = 0.013), farm category (p = 0.000), and experience in farming (p = 0.003). However, the remaining variables did not show any statistically significant variation in this investigation (Table-6).

**Table-6:** Multivariable and univariable analyses demonstrating the relationship between demographic variables and the level of attitudes, n = 228.

Variables	Attitude level		Univariable analyses		Multivariable analyses	
		
	Positive (%)	Negative (%)	OR (95% CI)	p-value	AOR (95% CI)	p-value
Gender						
Female	86	52	2.72 (1.57–4.71)	0.000	2.41 (1.19–4.87)	0.014
Male	34	56	Ref.		Ref.	
Age						
31–40 years	60	18	8.00 (3.23–19.80)	0.000	1.38 (0.23–8.08)	0.419
41–50 years	37	35	2.53 (1.06–6.06)		0.43 (0.05–3.59)	
≥50 years	13	31	1.00 (0.37–2.68)		0.26 (0.02–2.68)	
18–30 years	10	24	Ref.		Ref.	
Education						
Primary	29	15	8.21 (3.05–22.13)	0.000	4.36 (0.96–19.79)	0.0013
Secondary	40	24	7.08 (2.81–17.80)		4.62 (1.37–15.58)	
Higher secondary	29	19	6.48 (2.47–16.99)		6.49 (1.95–21.64)	
Graduation and above	14	16	3.71 (1.29–10.65)		16.89 (2.00–14.19)	
No formal education	8	34	Ref.		Ref.	
District						
Rangpur	31	29	0.56 (0.26–1.21)	0.200		
Panchagarh	29	31	0.49 (0.23–1.06)			
Thakurgaon	26	30	0.45 (0.21–0.99)			
Dinajpur	34	18	Ref.
Farm type						
Goat	64	34	6.18 (2.98–12.81)	0.000	7.08 (2.95–16.97)	0.000
Mixed	42	28	4.92 (2.29–10.60)		5.45 (2.16–13.72)	
Sheep	14	46	Ref.		Ref.	
Experience in farming						
6–10 years	13	17	4.07 (1.31–12.65)	0.000	6.56 (4.19–16.18)	0.003
11–15 years	37	39	5.06 (1.89–13.49)		3.37 (3.17–20.84)	
>15 years	64	20	17.06 (6.24–46.67)		5.39 (4.96–32.56)	
1–5 years	6	32	Ref.		Ref.	
Training on livestock diseases and vaccination						
Received	22	30	0.58 (0.31–1.09)	0.092		
Not received	98	78	Ref.			

CI: Confidence interval, OR: Odd ratio, AOR: Adjusted odd ratio, Ref: Reference category

#### Practice of farmers

Only 22.8% (52/228) of the farmers participating in this study demonstrated a satisfactory level of practice regarding vaccine use (Figure-2c). Univariate analysis revealed significant associations (p < 0.05) between participants practice level and gender, district, farm category, and vaccine training. Female farmers exhibited 2.65 times greater odds of possessing better practices than males. Participants from the Thakurgaon region showed 1.54 times better practice (95% CI: 0.66–3.55) than those from the Dinajpur region. Goat farmers demonstrated significantly higher levels of good practice (OR: 3.97; 95% CI: 1.54–10.23) compared to sheep farmers. Surprisingly, farmers who received vaccination training from any organization were 85% less likely (OR = 0.15; 95% CI: 0.04–0.53) to demonstrate good practice than their non-trained counterparts. However, age, education, and experience in farming did not emerge as statistically significant variables in this survey (Table-7). The results of the multivariate analysis indicated that the practice level of SRF varied significantly according to gender (p = 0.021), districts (p = 0.042), farm category (p = 0.025), and training on vaccines (p = 0.004). Nonetheless, the remaining variables did not show any statistically significant variation in this investigation (Table-7).

**Table-7:** Multivariable and univariable analyses demonstrating the relationship between demographic variables and the level of practices, n = 228.

Variables	Practice level		Univariable analyses		Multivariable analyses	
		
	Good (%)	Poor (%)	OR (95%CI)	p-value	AOR (95%CI)	p-value
Gender						
Female	40	98	2.65 (1.30–5.39)	0.007	2.43 (1.14–5.18)	0.021
Male	12	78	Ref.		Ref.	
Age						
31–40 years	7	27	1.33 (0.50–3.52)	0.800	
41–50 years	20	58	1.19 (0.44–3.21)			
≥50 years	17	55	0.85 (0.27–2.65)			
18–30 years	8	36	Ref.		
Education						
Primary	9	35	3.34 (0.83–13.34)	0.095		
Secondary	20	44	5.90 (1.63–21.41)			
Higher secondary	13	35	4.82 (1.27–18.36)			
Graduation and above	28	40	3.95 (0.93–16.82)			
No formal education	7	23	Ref.
District						
Rangpur	7	53	0.39 (0.14–1.08)	0.049	0.37 (0.12–1.08)	0.042
Panchagarh	13	47	0.83 (0.34–1.99)		0.79 (0.30–2.05)	
Thakurgaon	19	37	1.54 (0.66–3.55)		1.61 (0.65–4.00)	
Dinajpur	13	39	Ref.		Ref.	
Farm type						
Goat	30	68	3.97 (1.54–10.23)	0.016	3.76 (1.39–10.17)	0.025
Mixed	16	54	2.66 (0.97–7.33)		2.15 (0.74–6.24)	
Sheep	6	54	Ref.		Ref.	
Experience in farming						
6–10 years	6	24	2.12 (0.54–8.35)	0.243		
11–15 years	20	56	3.03 (0.95–9.63)			
>15 years	22	62	3.01 (0.96–9.47)			
1–5 years	4	34	Ref.
Training on livestock diseases and vaccination						
Received	3	49	0.15 (0.04–0.53)	0.003	0.15 (0.04–0.54)	0.004
Not received	49	127	Ref.		Ref.	

CI: confidence interval, OR: odd ratio, AOR: Adjusted odd ratio, Ref: reference category

### Associations between farmer’s KAP

Table-8 shows that the KAP scores were positively correlated according to the Spearman’s rank correlation test. There was a significant relationship of 0.64 (p < 0.001) between the knowledge and attitude scores. Likewise, there was a correlation of 0.61 (p < 0.001) between knowledge and practice scores. Attitude and practice had the lowest correlation coefficient of 0.47 (p < 0.001). Strong positive correlations were found between knowledge and attitude; between knowledge and practice; and between practice and attitude [24].

**Table-8:** Correlations between KAP on vaccine usage (p ≤ 0.001).

Variables	Correlation coefficient	p-value
Knowledge-Attitudes	0.649	0.000
Knowledge-Practices	0.616	0.000
Attitudes-Practices	0.474	0.000

KAP: Knowledge, attitudes, and practices

## Discussion

### Knowledge of farmers

This study is the first to assess the SRF’s KAP toward vaccine usage in Bangladesh. In addition, we examined determinants of total KAP levels among the study group. Our research reveals both coincidences and disparities with previous studies by Robi *et al*. [12] and Girma *et al*. [20] on livestock vaccine KAP. Our investigation determined that farmers had an average knowledge level of vaccine usage at 42.1% (Figure-2A), similar to findings in Oromia, Ethiopia [20], but less than the rate in a separate study in Southwest Ethiopia [12]. About 59.6% of farmers exhibited a moderate understanding of small ruminant diseases similar to earlier findings [8, 16]. Historical context plays a crucial role in disease control, as demonstrated by previous research by Win *et al*. [15] and Nuvey *et al*. [25]. Previous epidemics have had a significant impact on farmers’ understanding of vaccination. Effective disease control relies on prioritizing diseases for livestock vaccination [6]. Prioritized vaccine targets in Bangladesh have been identified as PPR, goat and sheep pox, foot and mouth disease, hemorrhagic septicemia, black quarter, anthrax, and rabies [5, 10]. Our investigation found that 41.7% of participants knew about this priority small ruminant immunization, aligning with the latest Ethiopian survey [6]. Our survey revealed that most farmers hold a negative perception of the health benefits associated with small ruminant vaccination (Figure-2), corroborating findings from southwest Ethiopian research [12]. Disseminating evidence-based information on the advantages of livestock vaccines, such as reduced disease rates and improved animal well-being, could alter this perception [20, 25, 26]. Farmers who did not vaccinate their livestock reported no challenges [27]. About 38.6% of our survey participants believe that small ruminant diseases can be managed without vaccinations. To improve farmers’ understanding and acceptance of livestock vaccines, it is essential to educate them about the program’s objectives and benefits [9, 28]. About 39.5% in our research concur with the most recent study that vaccines can shield small ruminants against diseases [12]. About 74.6% of SRF in the study believed that vaccines are effective in preventing uncommon diseases with minimal impact on overall health. The previous studies in southwest Ethiopia [12] and Ormia, Ethiopia [20] also reported similar results. Vaccines help curb the spread of antimicrobial resistance by reducing the demand for antibiotic therapy. Adopting vaccination protocols helps prevent zoonotic diseases [29]. A significant proportion of farmers in our study held the view that routine vaccinations could alleviate antibiotic resistance concerns, as shown in Table-2. Zoonotic diseases could be prevented through the perception of vaccination as an effective tool. Farmers saw vaccination as the only remedy for particular diseases.

Participation of women in farmers’ training sessions for livestock vaccination use is culturally discouraged [30]. Goat farming experience for 6–10 years among female participants with a graduate degree was linked to a greater degree of vaccine knowledge, but less so if they had attended vaccination training. Although farmers’ training is crucial for establishing a baseline understanding of livestock vaccine use, female participation in meetings, training sessions, and other activities in the study area is culturally uncommon [30].

### Attitudes of farmers

Vaccination protects livestock productivity and the health of animals and humans by preventing diseases [20]. To boost livestock vaccination rates, it may be necessary to revise the disease monitoring system and ensure better vaccine accessibility [31]. According to a previous study by Robi *et al*. [12], and as shown in Table-3, nearly half (50.9%) of the participants were knowledgeable about the existence of vaccines for small ruminant diseases. The cost of small ruminant vaccines affected participants’ attitudes, aligning with results from related studies by Robi *et al*. [12] and Habiyaremye *et al*. [32]. The economic significance of financial factors in vaccinating livestock is underscored [9, 33]. Like studies by Robi *et al*. [12] and Habiyaremye *et al*. [32], our study found a connection between participants’ vaccine usage and their views on government funding for small ruminant vaccinations. Vaccination not only secures livestock productivity but also safeguards the health of both animals and humans by preventing disease outbreaks [20]. Most farmers consider vaccinations to enhance animal and human safety while boosting small ruminant production and well-being. The use of vaccines in food-producing animals lessens the demand for antibiotics [34]. Vaccines eradicate disease-causing organisms while bolstering herd immunity [35]. Although the belief contradicts scientific consensus, many vaccines necessitate booster doses for optimal and enduring immunity [36]. About 38.2% of small ruminant participants held the belief that a single vaccination would provide them with perpetual immunity, consistent with previous studies in southwest Ethiopia [12]. However, this belief contradicts the scientific consensus, which suggests that many vaccines require booster doses for optimal and long-lasting immunity [36]. About 40.8% of our study participants agree that one vaccine guarantees protection for all diseases, as indicated in a previous study by Robi *et al*. [12]. Despite this, participants in our study recognized the necessity of multiple vaccines to safeguard small ruminants from various diseases, influencing their attitudes toward the feasibility and effectiveness of vaccination programs [34]. By reducing livestock mortality, vaccination support programs effectively boost food security [37]. Some farmers in our study, as shown in Table-3, recognized the importance of small ruminant vaccinations for food safety. The correlation between participants’ opinions and individually vaccinating small ruminants for complete herd protection was significant. Vaccinating livestock is essential to attaining the United Nation’s Sustainable Development Goals [16]. Some farmers believe that vaccinations contribute to the long-term viability of small ruminant farming.

The present investigation, similar to previous research by Robi *et al*. [12] and Girma *et al*. [20] reveals that attitudes toward small ruminant vaccines significantly differ based on sociodemographic factors, such as gender, education, farm type, and farming experience (Table-6). Among female farmers with advanced degrees and 6–10 years of experience in goat farming, particularly positive attitudes toward small ruminant vaccines are observed. The attitude and knowledge levels exhibited a strong positive correlation. The relationship between attitudes and knowledge level may account for this correlation, aligning with previous research by Robi *et al*. [12] and Girma *et al*. [20]. Training does not always result in positive attitudes, unlike knowledge levels which are consistently linked to them.

### Practice of farmers

Perceived vaccine effectiveness, personal experiences, and confidence levels in veterinary knowledge could account for the disparate views among individuals on vaccination [12]. The figure (Figure-2) shows that the average practice score of 22.8% was lower than both the knowledge (42.1%) and attitude (52.6%) scores. About 61.8% of the farmers in our study vaccinated their small ruminants, higher than prior research in Bangladesh [5, 17, 18] and southwest Ethiopia [12]. Participants’ vaccination practices significantly influenced the timing of small ruminant vaccinations. While some farmers consult veterinarians, others rely on advice from fellow farmers or wait until disease outbreaks (Table-4). These differences in perspectives may stem from variations in perceived vaccine effectiveness, personal experiences, or confidence levels in veterinary knowledge [12]. About 43.4% of farmers in our study kept vaccination records, aligning with the results of previous research by Hossain *et al*. [30], Habiyaremye *et al*. [32], and Subedi *et al*. [38]. Although our study revealed that just 40.4% of farmers practiced following vaccination schedules, this is notably less than the 79% reported in a Bangladesh study by Tasmim *et al*. [39]. Uneven application of practices can trigger disease outbreaks anywhere and anytime. The widespread use of vaccines as the most effective strategy for preventing small ruminant diseases is limited by an insufficient vaccine supply [9]. About 39.0% of participants in this study reported a higher rate of certain vaccine unavailability compared to previous studies by Williams *et al*. [9], Sultan *et al*. [40], and Ratan *et al*. [41]. Less than one-third (30.3%) of the participants exhibited inappropriate practices by neglecting to read the vaccine bottle prospectus, which is a lower percentage than that in the research conducted in Bangladesh [30]. Effective vaccine storage ensures a successful vaccination program [42]. Concerningly, most farmers in our study neglect to routinely check vaccine expiration dates or dispose of used or expired vials properly. Vaccinating farm animals correctly is vital for preventing diseases on the farm. While our findings reveal that just one-third (33.8%) of farmers have adequately immunized their small ruminants, this fraction is smaller than the corresponding Bangladeshi report [40]. To ensure vaccine effectiveness, they should be stored between 2°C and 8°C [43]. Proper storage of vaccines is essential for the success or failure of any vaccination program 44]. Smallholder livestock farmers in South Africa and Cambodia face challenges with vaccine storage, often storing vaccines in the same fridge as food, posing a risk of food contamination and accidental ingestion by children [32, 44]. Consequently, 31% of farmers in South Africa refused refrigerated vaccines for safety reasons, whereas 19% were unsure [32]. About 32.9% of farmers in the study stored vaccines in multipurpose fridges alongside food items (Table-4). Comprehensive training and awareness programs are essential to prevent hazards from farming practices.

Farmers’ knowledge and attitudes usually have a positive influence on practice [45]. Sociodemographic factors, including gender, district, farm type, and training, significantly impact farmers’ vaccination practice scores (Table-7). Female goat farmers in Thakurgaon district exhibit better vaccination habits than their counterparts, though fewer have received formal vaccination training. In our study, the impact of educational background on practice scores was insignificant. This is surprising, as earlier research indicated that higher education levels resulted in better practice [12, 38, 46]. The insufficient practice levels observed in our research may account for the discrepancy, implying that educational interventions only impact knowledge and attitude, not practice, without adequate methods and awareness.

### Relationship between KAP

Enhancing farmers’ understanding of livestock vaccines could result in more favorable attitudes and improved practices. The study confirmed a significant and statistically positive correlation between KAP, with knowledge influencing both attitude and practice (Table-8). This correlation aligns with previous Bangladeshi research by Kalam *et al*. [22] and Sultan *et al*. [40]. These three elements collaborate to enhance immunization in farms.

### Limitations

It’s important to acknowledge the present study’s limitations. The self-reported data’s accuracy might have been affected by technique, social desirability, and memory recall biases. The study’s cross-sectional nature does not allow for causal conclusions. The small sample size may not accurately reflect the situation of KAP among Bangladesh’s small ruminant producers beyond the northern region. These findings can inform targeted initiatives to encourage proper vaccine usage and manage small ruminant diseases in Bangladesh, for stakeholders and policymakers.

## Conclusion

This study is the first KAP among SRF regarding vaccine use in Bangladesh. The results show that 42.1% have good knowledge and 52.6% have a positive attitude, and 22.8% exhibited poor practice regarding vaccinating small ruminants. The KAP implementation among farmers reveals an existing gap. Sociodemographic factors, including gender, education level, district, farm category, farming experience, and training were identified as key determinants of farmers’ KAP toward the utilization of vaccines. To improve farmers’ capabilities in this region, interventions are necessary. Approaches such as educational training initiatives for SRF could promote awareness and enhance good vaccination practices. The study underscores the significance of well-implemented immunization programs for enhancing the health and welfare of small ruminants. Further research is required to ensure that livestock communities are moving forward in the right direction.

## Authors’ Contributions

MSI and AKM: Conceived and designed the study. MSI, AKM, MRA, TI, OI, AY, MAAM, AKMZH, MB, JHT, YM, MR, and MAI: Performed the study, collected and analyzed data, interpreted the results, and drafted and revised the manuscript. MSI, AKM, MRA, and TI: Participated in scientific discussion. All the authors have read, reviewed, and approved the final manuscript.
